# Region-Specific Expression of Mitochondrial Complex I Genes during Murine Brain Development

**DOI:** 10.1371/journal.pone.0018897

**Published:** 2011-04-27

**Authors:** Stefanie Wirtz, Markus Schuelke

**Affiliations:** Department of Neuropediatrics and Department “Developmental Disorders of the Brain”, NeuroCure Clinical Research Centre, Charité Universitätsmedizin Berlin, Berlin, Germany; Brigham and Women's Hospital, Harvard Medical School, United States of America

## Abstract

Mutations in the nuclear encoded subunits of mitochondrial complex I (NADH:ubiquinone oxidoreductase) may cause circumscribed cerebral lesions ranging from degeneration of the striatal and brainstem gray matter (Leigh syndrome) to leukodystrophy. We hypothesized that such pattern of regional pathology might be due to local differences in the dependence on complex I function. Using *in situ* hybridization we investigated the relative expression of 33 nuclear encoded complex I subunits in different brain regions of the mouse at E11.5, E17.5, P1, P11, P28 and adult (12 weeks). With respect to timing and relative intensity of complex I gene expression we found a highly variant pattern in different regions during development. High average expression levels were detected in periods of intense neurogenesis. In cerebellar Purkinje and in hippocampal CA1/CA3 pyramidal neurons we found a second even higher peak during the period of synaptogenesis and maturation. The extraordinary dependence of these structures on complex I gene expression during synaptogenesis is in accord with our recent findings that gamma oscillations – known to be associated with higher cognitive functions of the mammalian brain – strongly depend on the complex I activity. However, with the exception of the mesencephalon, we detected only average complex I expression levels in the striatum and basal ganglia, which does not explain the exquisite vulnerability of these structures in mitochondrial disorders.

## Introduction

Mitochondria play an important role in energy metabolism and thus also affect brain development and neuronal growth. Dysfunction of the mitochondria is the pathophysiological basis of a continuously growing group of heterogeneous diseases and clinical syndromes, most of them associated with malfunction of the respiratory chain [1]–[3]. Mitochondrial disorders occur with an overall incidence of 13.1/100,000 live births [4] and patients generally suffer from a multi-system disorder involving mainly organs with high energy demand [5]. The clinical picture of respiratory chain defects is diverse, ranging from lethal neonatal disease to adult-onset neurodegeneration [6]–[8]. During infancy one of the most frequent clinical phenotypes is Leigh syndrome, a progressive, neurodegenerative disorder of the subcortical gray matter. It is characterized by bilateral symmetric brain lesions, particularly in the basal ganglia, putamen, thalamus, mesencephalon and brainstem [9]. Patients suffer from ataxia, seizures, nystagmus, dysphagia and central apneas. Other children are born healthy and develop progressive leukodystrophy later in life [10], [11]. One of the most common biochemical defects of mitochondria leading to Leigh syndrome is isolated complex I deficiency [5], [12]. Complex I (NADH:ubiquinone oxidoreductase), the largest multi-enzyme complex of the mitochondrial respiratory chain, transfers electrons from NADH_2_ to ubiquinone and uses the free energy to pump protons from the mitochondrial matrix into the inter-membranous space [13]–[15]. Human complex I consists of 45 structural subunits; seven of which are encoded by the mitochondrial DNA (mtDNA). Complex I has an L-shaped outline consisting of a hydrophobic membrane arm that is embedded in the inner mitochondrial membrane and a hydrophilic peripheral arm protruding into the mitochondrial matrix [14], [16]. The subunits can be sub-fractionated into three groups: the flavoprotein fraction (FP) which is responsible for FMN and NADH_2_ binding; the iron-protein fraction (IP), participating in a chain of redox reactions and the hydrophobic fraction (HP), which is important for proton translocation and the anchorage of complex I in the inner mitochondrial membrane [17], [18]. 14 subunits are highly conserved during evolution, have bacterial homologues and are thus considered as “core” units of complex I. They are indispensible for the basic catalytic function of the enzyme complex and comprise all mtDNA encoded subunits and seven nuclear encoded subunits, which contain the redox groups and most components of the proton translocation machinery [19], [20]. The exact function of the remaining accessory subunits is largely unknown. They likely play a role in organization and stabilization of the holoenzyme [15], [21]. Mutations leading to complex I deficiency affect mtDNA and nuclear encoded structural subunits [8], [22], [23] as well as assembly genes such as *NDUFAF2*, *C20RF7*, *NDUFAF3, NDUFAF4, NUBPL and FOXRED1* [24]–[27].

Leigh syndrome becomes clinically apparent during the first two years of life [28], but respiratory chain deficiency may even manifest antenatally [29]. Characteristic neuropathological features of mitochondrial disorders are often region-specific; such as hypoplasia of the *Corpus callosum* in pyruvate dehydrogenase complex (PDHc) deficiency or bilateral lesions of the brainstem, striatum and cerebellum in complex I deficiency [30]. We wondered whether such characteristic lesional patterns could be explained by the tissue specific time course of gene expression for important functional components of the respiratory chain during the embryonic-fetal period. Little is known about the antenatal gene expression of respiratory chain components in humans or animals. On the enzyme level Minai *et al.* (2008) investigated various tissues of aborted human fetuses for the activity of respiratory chain activities of the complexes I-V. They found that already at early stages of fetal development the respiratory chain complexes are enzymatically functional, although the absolute activities were lower than after birth [31]. Several animal studies also indicate an up-regulation of mitochondrial biogenesis and gene expression in the postnatal period [32], [33]. In contrast to these mainly biochemical investigations and global gene expression studies, the time course and regional specificity of respiratory chain gene expression during embryonic development had not been investigated before. We thus set out to investigate by *in situ* hybridization (ISH) whether the pattern of gene expression for complex I subunits in mice correlates with the pattern of neuropathology and brain dysfunction seen in human patients with complex I deficiency.

## Materials and Methods

### Tissue preparation

8-week-old C57BL/6J mice (Charles River, Sulzfeld, Germany) were set up for natural timed mating and the morning when a vaginal plug was present was counted as E0.5. Pregnant females were killed by atlantoaxial dislocation at E11.5 and E17.5 and the embryos were recovered. Postnatal animals were killed by decapitation at P1 and P11, and by atlantoaxial dislocation at P28 and adult. The brains were immediately removed, rinsed in PBS (137 mM NaCl; 2.7 mM KCl; 4.3 mM Na_2_HPO_4_; 1.4 mM NaH_2_PO_4_; pH 7.4) and stored at −80°C. Axial cryosections of 20 µm thickness were prepared on a cryostat and thaw-mounted on Superfrost® slides (Menzel, Braunschweig, Germany). Slides were stored at −20°C until use for ISH staining as well as for DAPI counterstaining of the nuclei. Animals were housed, kept and killed in accordance with the recommendations of the European Commission and permission was granted by the Berlin Animal Ethics Committee under the reference number LaGeSo G0040/05.

### Preparation of DIG-labeled RNA probes

Total RNA was extracted from mouse tissue using TRI-Reagent® (SIGMA-Aldrich, Taufkirchen, Germany). cDNA was prepared by reverse transcription using ThermoScript® RT-PCR System (Invitrogen, Karlsruhe, Germany) and was used to generate approximately 300 bp templates for ISH probes corresponding to the cDNA sequence of the according complex I genes. The DNA fragments were amplified by PCR using the proof-reading Phusion® DNA Polymerase (Finnzymes, Espoo, Finland) according to the manufacturer's instructions. The PCR-products were purified by gel filtration, sub-cloned into pGEM®-T Easy *via* a TA-cloning protocol (Promega, Mannheim, Germany) and controlled by automatic sequencing. For *in vitro* transcription from the T7 or SP6 promoter 30 µg of each DNA template was linearized and contaminating RNAses removed by subsequent phenol-chloroform extraction, ethanol precipitation and re-suspension in DEPC water. 1 µg of this DNA was then used as a template to generate DIG-labeled riboprobes with the DIG RNA Labeling Kit (Roche, Grenzach-Wyhlen, Germany) with T7 or SP6 RNA polymerase to generate the antisense strands depending on orientation of the template. After DNase treatment the reaction was stopped with EDTA and the labeled product was ethanol-precipitated and re-suspended in 12 µl DEPC-H_2_O.

### *In situ* hybridization

Slides were thawed at room temperature for 20 min and then fixed for 10 min in 4% DEPC-paraformaldehyde. The slides were washed thrice in DEPC-PBS and then incubated in acetylation buffer (0.25% acetic anhydride in 0.1 M triethanolamine, pH 8.0). After washing thrice in DEPC-PBS, slides were pre-hybridized for 4 h with 700 µl hybridization buffer (50% form; 5x SSC; 5x Denhardt's solution; 150 µg/ml tRNA; 50 µg/ml sperm DNA). Hybridization was performed overnight at 60°C using 150 µl hybridization buffer plus 1 µg/μl specific DIG-labeled RNA probe. The slides were washed with 5x SSC for 5 min at room temperature, with 0.2x SSC for 30 min at 60°C (4x) and with 0.2x SSC as well as B1 (0.1 M Tris, pH 8.0; 0.15 M NaCl) for 5 min at room temperature. The slides were blocked for 4 h at room temperature with 1 ml blocking buffer (5% normal goat serum in B1) and then treated with 150 µl anti-digoxigenin antibody from sheep, conjugated with alkaline phosphatase (1∶2,500) overnight at 4°C. After rinsing the slides thrice with B1 and once with NTMT (0.1 M Tris, pH 8.0; 0.1 M NaCl; 0.05 M MgCl_2_; 0,1% Tween) each for 5 min at room temperature, the enzymatic colorimetric reaction was performed for 24–48 h using BM Purple AP substrate (Roche, Grenzach-Wyhlen, Germany). The slides were counterstained with DAPI (1 mg/ml, 1∶2,000 in PBS) for nuclear labeling and mounted with Hydro-Matrix (Micro Tech Lab, Graz, Austria).

### Signal detection and quantification

**Figure 1:**
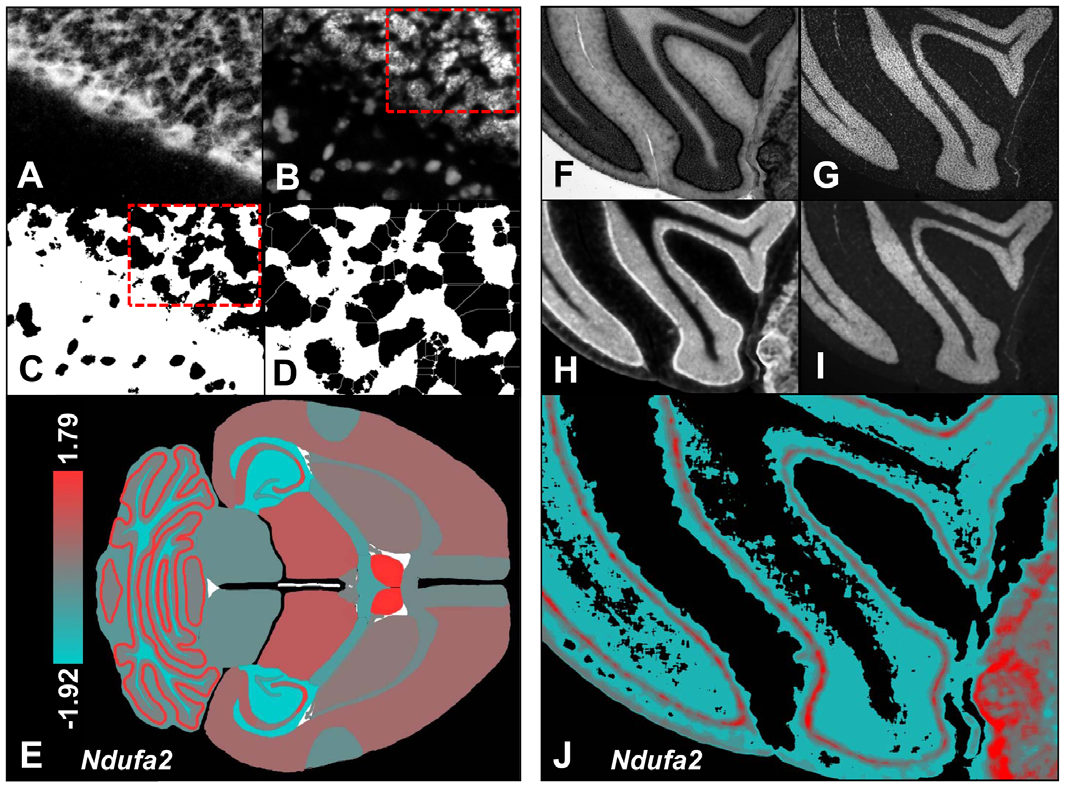
Densitometric quantitative analysis of the ISH signal and normalization to nuclear packing density. ***Normalization to the nuclear packing density:*** (**A**) Inverted image of the ISH signal from a DIG-labeled probe (*Ndufa2*) as used for local density integration, (**B**) Same ROI stained with a DNA specific nuclear stain (DAPI) for assessment of the nuclear packing density, (**C**) Image B after thresholding and image processing for automatic cell counting, (**D**) Results of the “watershed” algorithm (WCIF ImageJ v1.37a) enabling automatic discrimination of even partially overlapping signals from adjacent cells; image D corresponds to the red frame marked on B & C, (**E**) Heat scale image of site specific *Ndufa2* ISH signal per nuclear densities in the whole brain. The signal intensities are coded with heat scale colors (left). The numbers depict the extremes of the Z-transformed signal intensities in the cerebellum. ***Image analysis and normalization by image mathematics***. (**F**) Signal of the DIG-labeled *Ndufa2* ISH probe in the cerebellum, (**G**) Same ROI stained with DAPI, (**H**) Image F after inversion and density interpolation of 20 pixels, (**I**) Image G after density interpolation of 20 pixels, (**J**) Result of image calculation (division of image H by image I); the grey shades of the ensuing b/w image were re-coded with the same heat-scale colors as in E.

**Figure 2:**
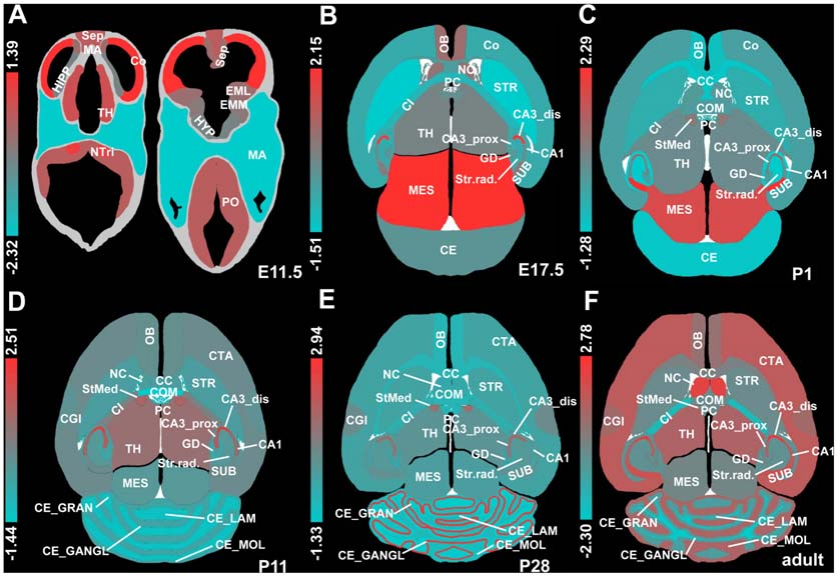
Arithmetic average of expression intensities of 33 structural complex I subunits during murine brain development. (**A–F**) Color coded average expression intensities for time points of prenatal [E11.5, E17.5] and postnatal [P1, P11, P28, adult (12 weeks)] development. The heat-scales depict the variation between the extremes of Z-transformed intensities (given as numbers); red color reflects high and blue color low expression levels. The abbreviations for the various brain structures and the developmental stage of their first investigation are given on Figure S1.

Conventional microscopic images were obtained with a cooled digital CCD-camera (SPOT3, Visitron, Puchheim, Germany) attached to an upright Leica DMLB microscope (Leica Microsystems, Wetzlar, Germany). Images were recorded with a x63 objective: ISH images under bright-field illumination and DAPI images under UV illumination at 380±10 nm excitation. All images were processed using ImageJ v1.14 software (http://rsb.info.nih.gov/ij/): b/w ISH images were inverted and the region of interest (ROI) was demarcated. The average densities in the respecting areas were measured by densitometry and the integrated densities were recorded. The integrated density of an unstained slide at identical illumination was subtracted as background density. To analyze and compare the relative expression intensities of ISH signals (ISH signal per nucleus) in different brain regions the ISH signal had to be normalized to the nuclear packing density, which was assessed in the DAPI images. For estimation of the relative signal intensity and nuclear packing density we used two alternative methods. On the one hand we performed an image analysis to estimate the nuclear packing density by direct counting of the cell nuclei either by manual counting or using the “watershed” algorithm of ImageJ, which was able to discern even between individual nuclei that lay in very close proximity or even partially overlapped (Figure 1A–E). For the embryonic stages, where the nuclei were too densely packed to discern between them, we used an alternative method and normalized the ISH signals to the nuclear package density by image mathematics (Figure 1F–J) *via* division of the ISH by the DAPI signal for each respective ROI. As ISH signals do not scale linearly, the relative signal intensities were Z-transformed and the Z-values were visualized as heat scale images. High Z-values signified high expression and were coded in red, low values in blue (Figure 1 and 2). Additionally, the expression patterns for all probes at all pre- and postnatal stages were depicted in clustergrams (Figure S2) as well as in heat scale images (File S1).

### Statistical analysis

**Figure 3:**
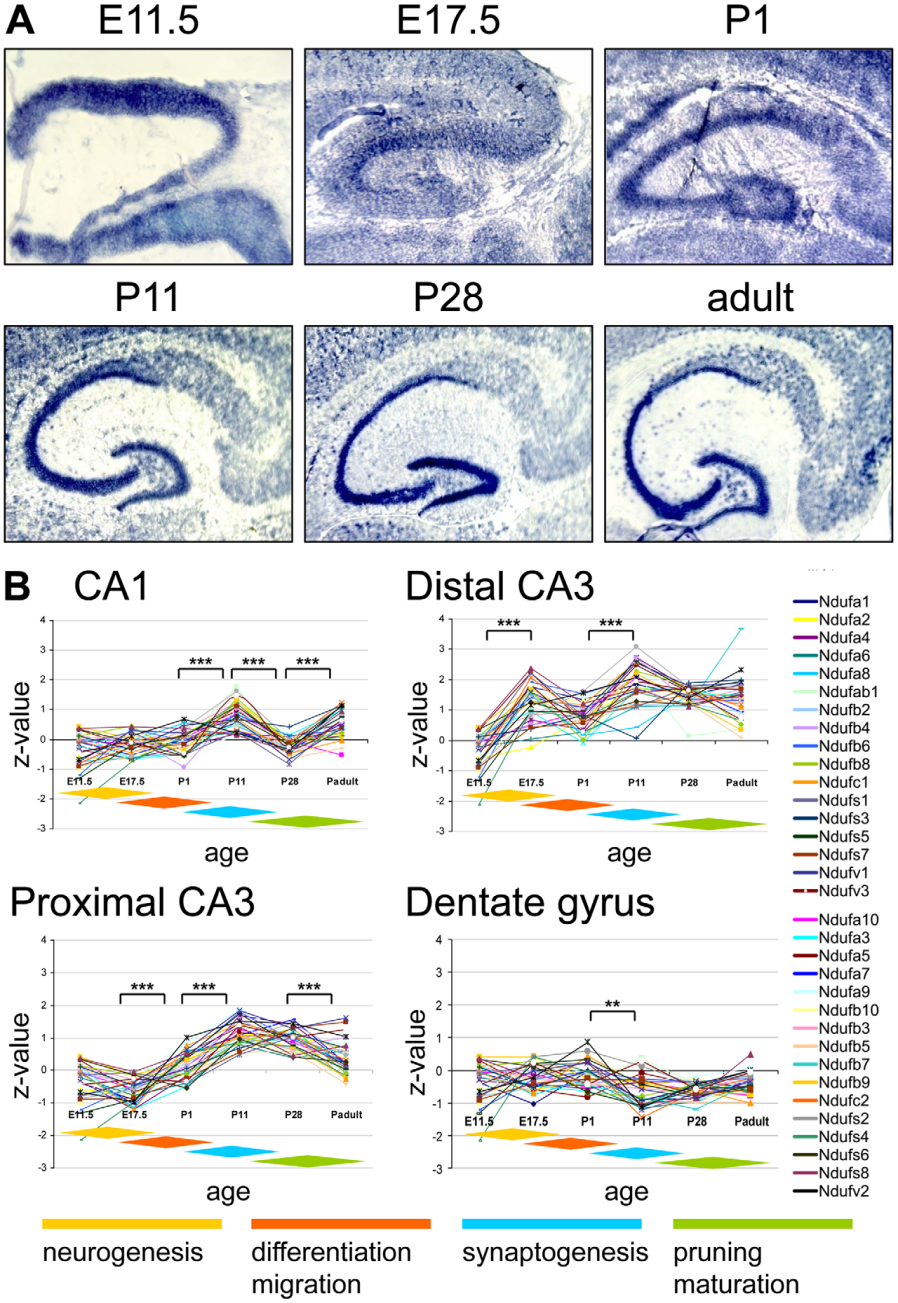
Complex I expression in the murine hippocampus. (**A**) ISH image for the complex I subunit *Ndufb2* probe in the hippocampus during pre- and postnatal development. The images were recorded at x10 magnification. At E11.5 the hippocampal subfields had not yet delineated, thus the expression was investigated for the entire hippocampal region (HIPP). (**B**) Z-transformed expression intensities for each probe plotted against the developmental age in four hippocampal subfields. The diamond shaped color fields illustrate the region specific stages of neuronal development. Significance levels of differences in expression are depicted by stars: *** p<0.0001; ** p<0.001.

The time course of all Z-transformed relative signal intensities was plotted against the age of the animal for each probe and brain region (Figures 3–6). As relative complex I expression intensities within an individual brain region were not normally distributed during development, we used the non-parametric Mann-Whitney U-test on the StatView v5.0 software (SAS Institute Inc., Cary, USA) to search for significant differences.

**Figure 4 pone-0018897-g004:**
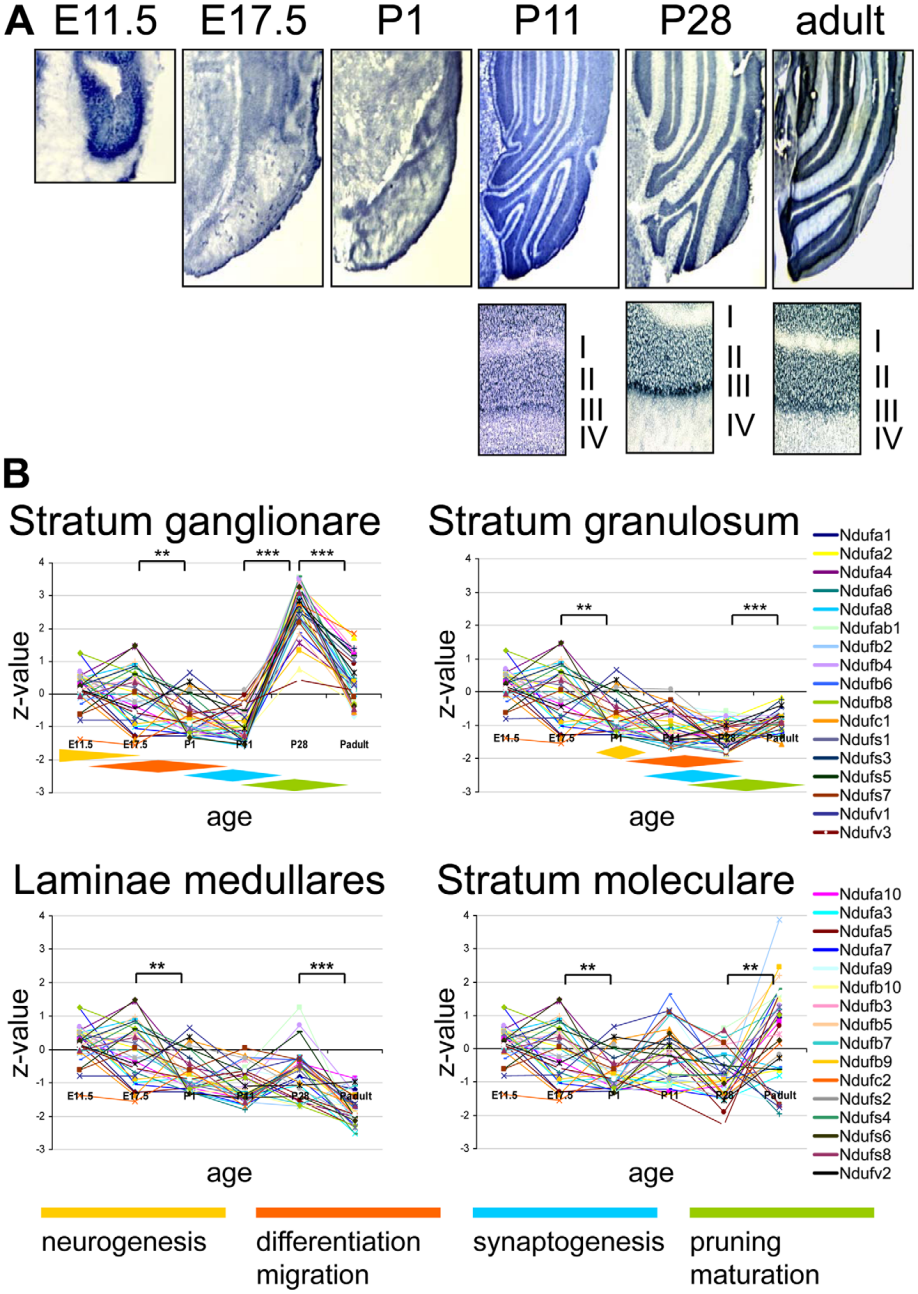
Complex I expression in the murine cerebellum. (**A**) ISH image for the complex I subunit *Ndufb2* probe in the cerebellum during pre- and postnatal development. The images were recorded at x2.5 and the detailed insets at x10 magnification. The roman numbers depict the cerebellar layers: (**I**) *Laminae medullares*, (**II**) *Stratum granulosum*, (**III**) *Stratum ganglionare*, Purkinje cells and (**IV**) *Stratum moleculare*. Between E17.5 and P1 gene expression is only recorded for the cerebellar field (CE) and before that at E11.5 at the dorsal border of the rhomboid fossa (RF) (**B**) Z-transformed expression intensities for each probe plotted against the developmental age in the four cerebellar strata or the cerebellar field. The diamond shaped color fields illustrate the region specific stages of neuronal development. Significance levels of differences in expression are depicted by stars: *** p<0.0001; ** p<0.001.

**Figure 5 pone-0018897-g005:**
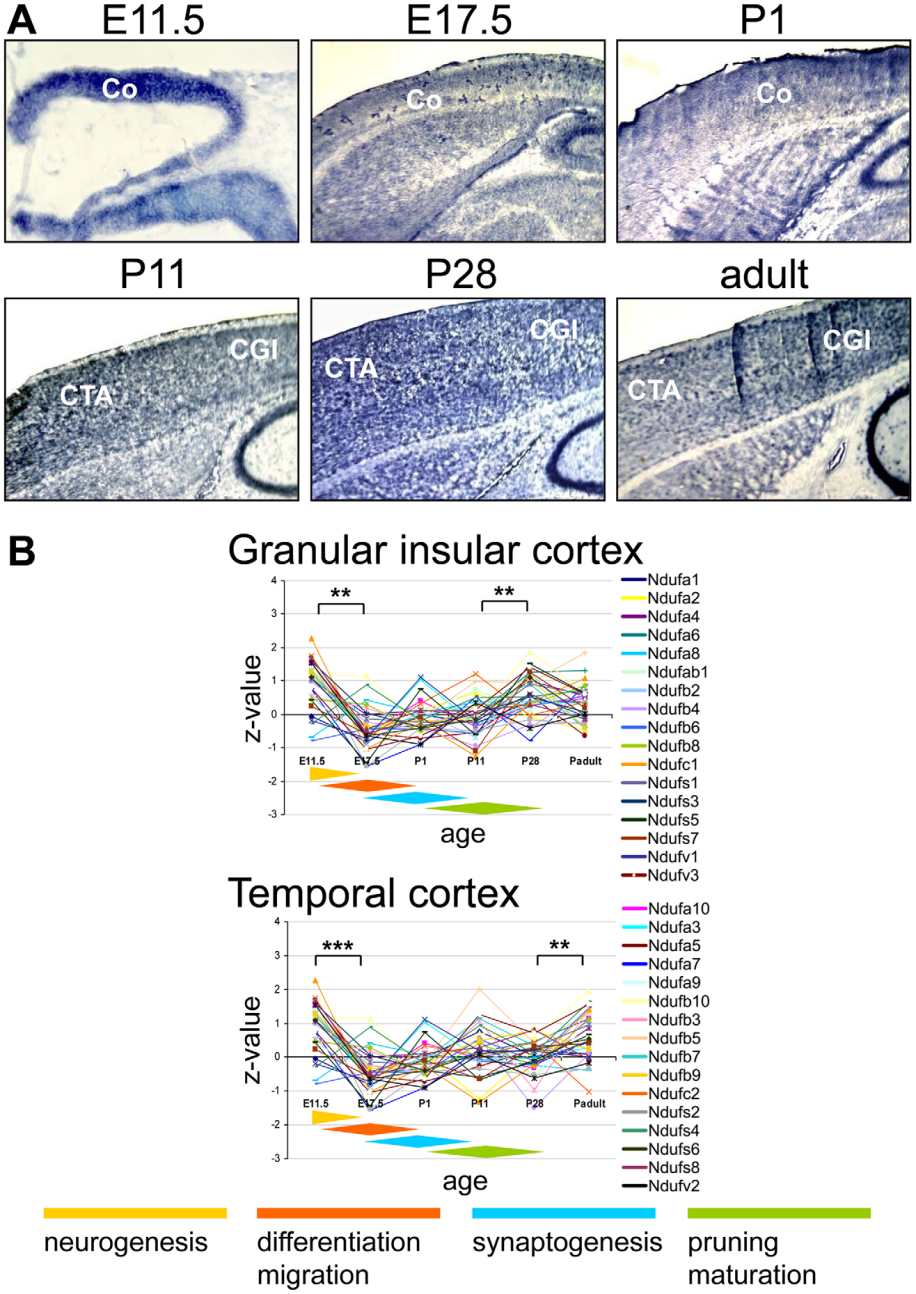
Complex I expression in the murine cerebral cortex. (**A**) ISH image for the complex I subunit *Ndufb2* probe in the cerebral cortex during pre- and postnatal development. The images were recorded with x10 at E11.5 and with x2.5 magnification thereafter. (**B**) Z-transformed expression intensities for each probe plotted against the developmental age in two sections of the cerebral cortex. The diamond shaped color fields illustrate the region specific stages of neuronal development. Significance levels of differences in expression are depicted by stars: *** p<0.0001; ** p<0.001; CGI, Cortex-granular insular; CTA, Cortex-temporal anterior.

**Figure 6:**
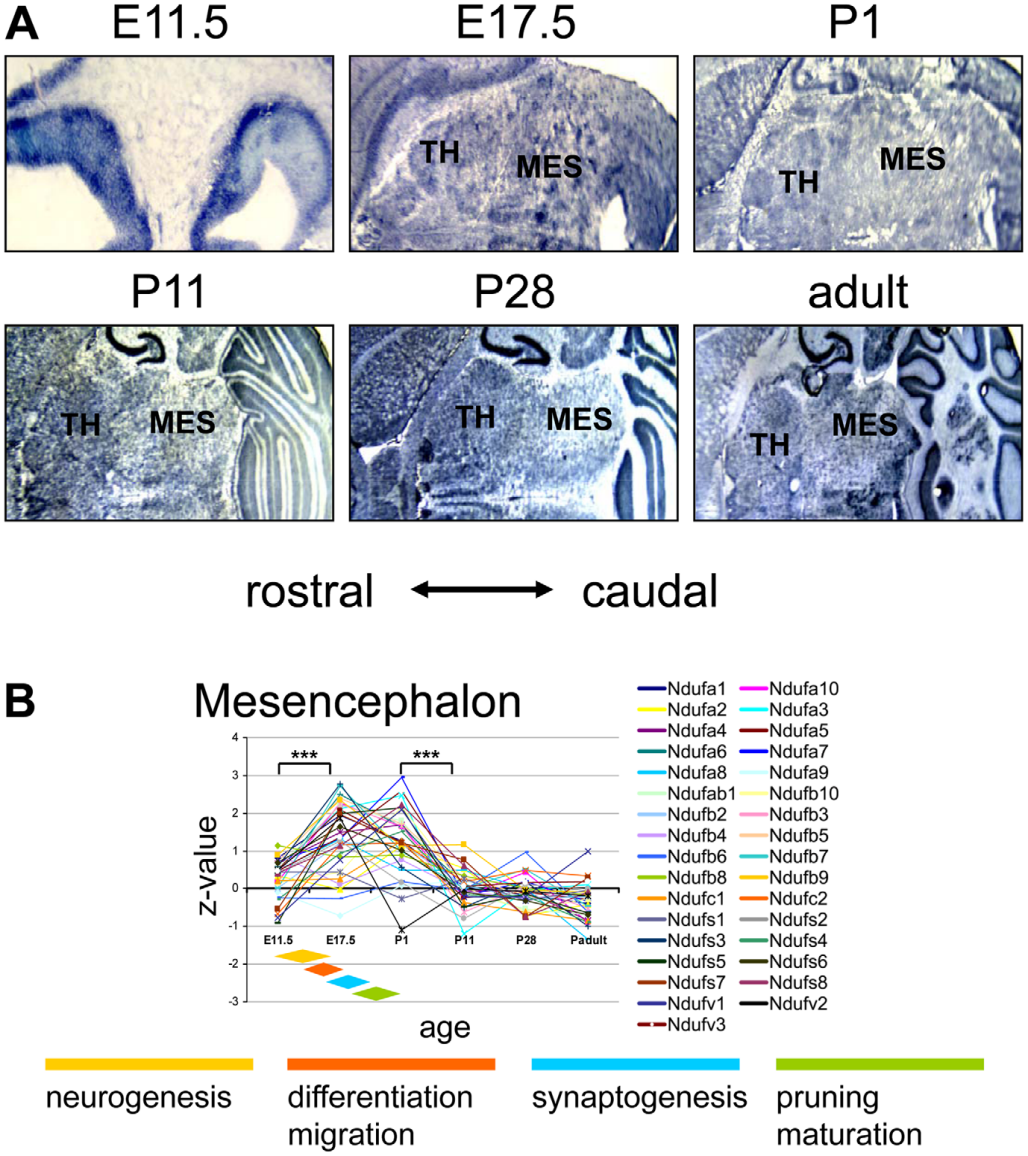
Complex I expression in the murine mesencephalon. (**A**) ISH image for the complex I subunit *Ndufb2* probe in the mesencephlaon during pre- and postnatal development. The images were recorded at x10 (E11.5) and at x2.5 magnification thereafter. At E11.5 the expression is shown in the precursor structures of mesencephalon pons and thalamus. (**B**) Z-transformed expression intensities for each probe plotted against the developmental age in two sections of the cerebral cortex. The diamond shaped color fields illustrate the region specific stages of neuronal development. Significance levels of differences in expression are depicted by stars: *** p<0.0001; ** p<0.001; CGI, Cortex-granular insular; CTA, Cortex-temporal anterior. MES, mesencephalon; TH, thalamus.

## Results

### Differences in the relative expression intensities of complex I subunits during development

We examined by *in situ* hybridization the expression patterns of all known nuclear encoded structural subunits of mouse complex I in different brain regions at the six developmental stages E11.5, E17.5, P1, P11, P28 and adult. Due to the supposed natural variation of expression intensity of single subunits during a onetime measurement coupled with the imprecision of semi-quantitative densitometry, we here only considered for statistical analysis the joint average expression levels (arithmetic average) of all nuclear encoded subunits. We found a highly regionalized expression pattern for complex I subunits, which varied after Z-transformation up to four standard deviations between different brain regions. The average densities of all 33 probes are depicted as heat scale images on Figure 2 for all investigated stages of pre- and postnatal brain development. For detailed visual rendition of individual probe densities we provide clustergrams (Figure S2) and heat scale images (File S1).

On Figures 3–6 we highlight the expression patterns of complex I subunits in those brain regions that are mainly affected by mitochondrial disorders (hippocampus, cerebellum, cortex and mesencephalon). Below each diagram we provide the region-specific milestones of neuronal development such as **(1)** neurogenesis, **(2)** differentiation/migration, **(3)** synaptogenesis, and **(4)** pruning/maturation.

### Complex I gene expression in the hippocampus

In the hippocampus we found a highly specific expression pattern during development and analyzed four hippocampal subfields: the CA1, distal and proximal CA3 regions and the dentate gyrus (Figure 3). Beyond embryonic day E11.5 the highest intensities were found in the distal CA3 region, followed by the proximal CA3 and the CA1 region. The relative expression intensities of all three regions of the *Cornu ammonis* peak at P11 (p<0.0001) and coincide with the time point of most intense synaptogenesis [34]–[36]. Beyond that, at embryonic day E17.5 we found an additional peak only in the distal CA3 region coinciding with a period of intense neurogenesis [34]–[36]. The dentate gyrus in contrast had a completely different expression pattern with low relative expression intensities and without significant variation of expression levels during development, except for a slight drop between P1 and P11 (p<0.001; Figure 3).

### Complex I gene expression in the cerebellum

In the cerebellum we investigated the *Stratum ganglionare* (Purkinje cells), *Stratum granulosum* (granule cells), *Laminae medullares* and *Stratum moleculare* (molecular layer) (Figure 4). As these distinct cerebellar substructures do not demarcate before postnatal day P1, we only refer to the cerebellar field (CE) before that (for naming conventions see Figure S1). In the Purkinje cell layer the expression levels peak at P28, which coincides with synaptogenesis and maturation of the cerebellum [37]–[40]. Interestingly, at this time point these were the highest relative intensities not only in the cerebellum but in the entire brain, even if compared to the distal CA3 region of the hippocampus. In contrast, the expression levels within the granule cells and the *Laminae medullares* continuously dropped as these structures demarcated themselves from the undifferentiated cerebellar field and remain the lowest in the entire brain. The expression intensities of the molecular layer remain largely constant and due to the low number of cell bodies the expression intensities of the different probes vary widely.

### Complex I gene expression in the cerebral cortex

In the granular insular and the temporal cortex we found the highest expression intensities at the earliest stages of cerebral development at E11.5 (Figure 5), which were then the highest of all investigated brain regions. After that the relative expression levels declined and were subject to only minimal variation until the adult stage.

### Complex I gene expression in the mesencephalon

The expression intensities of complex I subunits in the mesencephalon peaked at the late embryonic and early postnatal stages. The highest relative expression intensities were found between E17.5 and P1. At E17.5 the relative complex I mRNA levels in the mesencephalon are the highest ones detected of the entire brain. Soon after birth signal intensities decline and remain constant until adulthood (Figure 6).

## Discussion

The expression levels of the 33 nuclear encoded subunits of murine mitochondrial complex I undergo drastic changes during development depending on the examined brain region. Neuronal development of the brain proceeds through the stages of **(1)** neurogenesis, **(2)** neuronal differentiation/migration, **(3)** synaptogenesis and **(4)** pruning/maturation, while the timing of these stages largely differs between individual brain regions [41]. In order to cover important developmental stages of various brain regions, we had chosen the time points E11.5, E17.5, P1, P11, P28 and adult (12 weeks) for investigation.

### Quantification of ISH signals

In contrast to mRNA array analysis, ISH signals are notoriously difficult to quantify because numerous confounding factors might interfere [42]. On the other hand, ISH offers the advantage to assess gene expression at the cellular level. One problem is the non-linearity between signal strength and mRNA concentration. To address this question, Larsson and Hougaard (1994) have immobilized oligonucleotides on glass slides and with this “ideal” model system found a nearly logarithmic relationship between probe optical density and target oligonucleotide concentration over two to three orders of magnitude [43]. Additionally, one has to consider the different cellular packing densities in various brain regions, which varied in relative units from 0.381 (cerebellum, *Stratum moleculare*) to 4,453 (dentate gyrus) in the adult mouse. We thus counted the number of DAPI-stained nuclei in each ROI and divided its ISH signal intensity by the nuclear packing density. All corrected signal intensities from the same slide were then Z-transformed to overcome the problem of their non-linearity and absence of normal distribution, which finally enabled us to compare various brain regions on each slide. It is, however, important to keep in mind that ISH signals only reflect relative changes in copy numbers and thus only allow a relative assessment of the signal intensity in each section [42]. Our study would thus not be suited to detect an absolute overall increase in mitochondrial gene transcription after birth, as described by other scientists [31]–[33].

### Complex I gene expression in the hippocampus

Hippocampal neurons proliferate prenatally, followed by dynamic growth and differentiation around the neonatal period and synaptogenesis in the first postnatal week. In the second and third postnatal weeks synaptic connections are reinforced through triggered synaptic activity while the maturation is completed around the first month of life [34]–[36]. In the hippocampus we observed expression levels and dynamics that differed between (i) the CA regions and (ii) the dentate gyrus.

The high expression intensity in CA3, especially around postnatal day P11, might reflect a larger energy demand of the giant pyramidal neurons with their numerous synaptic contacts, which are the largest of the entire hippocampus and contrast with the smaller neuronal size of other hippocampal subregions [44]. Interestingly even in adult mice, exercise-induced genesis of dendritic spine synapses in the *Stratum radiatum* of the CA1 region strongly went in parallel with an up-regulation of mitochondrial number and oxidative capacity in those neurons [45]. Matching observations were made by Lein *et al.* (2004) who demonstrated *via* mRNA expression studies and high-throughput *in situ* hybridization that six out of seven exemplarily chosen genes involved in the hippocampal energy metabolism were highly enriched in CA3 [46]. Genome-wide mRNA expression studies in the developing hippocampus further discovered a high expression of genes encoding hippocampal proteins involved in glycolysis, oxidative metabolism and fatty acid synthesis. Amongst them were the complex I subunits Ndufb2 and Ndufs2, which were regulated in parallel with genes for synaptic function, signal transduction and ion channels [36]. Some of the complex I subunits are not exclusively involved in NADH_2_ oxidation and electron transport and provide a functional link to other energy generating pathways, e.g. Ndufab1 is related to Acyl-carrier proteins and might form a link between the respiratory chain and the *de novo* fatty acid synthesis [47]. An additional early expression peak in the distal part of CA3 at E17.5 correlates with a period of extensive neuronal proliferation in the hippocampus, which seems to set this subregion of the *Cornu ammonis* apart from their counterpart with respect to its developmental dynamics and energy metabolism.

The mRNA expression pattern of complex I genes in the dentate gyrus remained steady with only a slight decrease at P11 until the adult state. Whereas the *Cornu ammonis* regions mostly generate excitatory pyramidal neurons during development, the dentate gyrus consists of granule cells in whom low level neurogenesis persists throughout life [48]. Therefore, we probably did not find an expression peak that is restricted to a period of exclusive neurogenesis [44], [49].

### Complex I gene expression in the cerebellum

Cerebellar development starts with the neurogenesis of Purkinje cells around embryonic day E10.5. Their proliferation ceases shortly before birth, but in the postnatal period they still grow, mature and develop their synaptic network. The second prevalent cerebellar cell type is the granule cell. Their proliferation maximum is recorded in the first postnatal week followed by elongation and migration into the internal granular layer. During the third postnatal week the granule cells undergo synaptogenesis and further maturation [37]–[40]. In our study we found highly variant expression levels in the layers of the cerebellar cortex and different expression dynamics between Purkinje and granule cells.

In Purkinje neurons nearly all complex I subunits showed a sharp rise of expression intensity around postnatal day P28. In parallel to CA1 and CA3, the surge of complex I gene expression coincides with a period of intense synaptogenesis and synaptic plasticity in the second and third postnatal week. This interpretation is strengthened by life cell imaging experiments showing the importance of mitochondria in dendritic spines for the plasticity of synapses [50] and secondly by developmental gene expression studies of the whole cerebellum where parallel up-regulation was found for genes that are indispensible for synaptic activity and plasticity [51]. However, in contrast to whole cerebellum mRNA expression studies, ISH offers the possibility to study expression differences at the cellular level. Here we find a striking difference between the granule and Purkinje neurons, the latter possessing exceptionally large numbers of synaptic connections *via* their vast dendritic tree, which implies a high energy demand for the integration of excitatory and inhibitory synaptic inputs and subsequent maintenance of ion gradients. Such increased demand for mitochondrial function is also in line with a high spontaneous activity of these cells [52]. The importance of mitochondrial function for Purkinje cell development is further highlighted by *pcd* (Purkinje cell degeneration) mice that carry mutations in a mitochondrial zinc carboxypeptidase called Nna1. Homozygous mutant mice loose >99% of their Purkinje cells between postnatal days P15 and P35 [53]. This timing exactly corresponds with the up-regulation of complex I gene transcripts during postnatal development (Figure 4).

### Complex I gene expression in the cerebral cortex

Neurogenesis, neuronal differentiation and migration take place during prenatal development of the murine cerebral cortex. Postnatal development comprises axonal and dendritic growth, synaptogenesis and neuronal maturation [54]. In our study the highest expression levels were found at the initiation of cortical development around E11.5 which corresponds to a period of intense neurogenesis and then again later around the transition into adult life (Figure 5b). The complex I gene expression levels in the cortical regions are significantly lower than in CA3/CA1 and the Purkinje cell layer suggesting a more continous and prolonged process of synaptic maturation and plasticity.

### Complex I gene expression in the mesencephalon (midbrain)

The structures most often affected in Leigh syndrome and other mitochondrial disorders are the mesencephalon and brainstem [9]. Central structures are the mesodiencephalic dopaminergic (mDA) neurons of the *Substantia nigra,* which selectively degenerate in Parkinson's disease, and the red, pontine and olivary nuclei often affected in Leigh syndrome [30], [55]. Even in the course of pathogenic events leading to schizophrenia, considered to be a disorder of neurodevelopment [56], the interplay between the dopaminergic pathways and mitochondrial function seems to have a role as intracellular dopamine is able to suppress complex I activity in neuronal cells [57].

Midbrain and brainstem development starts early around E8.5 with the generation of mDA precursors. Embryonic days E10.5 and E12.5 see specification, early differentiation and migration and the late developmental period (E12.5–E14) is characterized by terminal differentiation, synaptogenesis and maturation [58]. Here we observed a high degree of complex I gene expression between embryonic day E17.5 and birth. Thereafter the expression levels declined and remained steady until the adult stage.

It is a well known fact that complex I inhibitors such as rotenone or MPP^+^ selectively harm mDA neurons and lead to Parkinson's disease [59]. The resulting loss of dopamine might be due to an energy-dependent disturbance in the pre-synapses of mDA neurons [60], [61]. This hypothesis is further strengthened by findings of an up-regulation of genes involved in oxidative phosphorylation and glycolysis after application of rotenone, probably in a compensatory fashion [62]. Given this selective sensitivity of mesencephalic and brainstem neurons to complex I inhibition or disruption might explain the high incidences of Leigh syndrome amongst patients with a genetic complex I deficiency [12].

### Conclusion

Contrary to our initial hypothesis assuming high complex I gene expression mainly in all those brain structures that are predominantly affected by Leigh syndrome, we only found this to be true for the mesencephalon. Gene expression in the striatum and the basal ganglia was average, did not vary much during development, and therefore does not provide an explanation for the exquisite vulnerability of these structures in states of complex I deficiency. However, we have to add the caveat that mRNA levels might not always reflect the amount of assembled complex I or the biochemical activity of the complex. With respect to timing and relative intensity of complex I gene expression, we found highly variant patterns in the developing mouse brain. High average complex I gene expression levels were detected during periods of intense neurogenesis [41]. A second even higher peak was present in Purkinje cells and hippocampal CA1/CA3 neurons during synaptogenesis and maturation, illustrating the fact that 81% of the cerebral energy consumption is due to action potentials and postsynaptic effects of glutamate [63]. These findings are in accord with a recent study of our group, in which we have shown that gamma oscillations in the hippocampus – often associated with higher cognitive functions of the mammalian brain – strongly depend on mitochondrial ATP production and especially on complex I function [64]. However, further studies are needed to elucidate the function of complex I in brain development and maintenance of its function, especially the striatum and the basal ganglia.

## Supporting Information

Figure S1 Investigated brain structures at the various time point of murine brain development. Abbreviations (*in alphabetic order*): CA1, CA1 region; CA3_dis, distal part of the CA3 region; CA3_prox, proximal part of the CA3 region; CC, *Corpus callosum*; CI, *Capsula interna*; CGI, cortex-granular insular; CE, cerebellar field; CE_GANGL, *Cerebellum stratum ganglionare*; CE_GRAN, *Cerebellum stratum granulosum*; CE_LAM, *Cerebellum laminae medullares*; CE_MOL, *Cerebellum stratum moleculare*; Co, cortex; COM, *Commissura anterior*; CTA, cortex-temporal anterior; EML, *Eminentia lateralis*; EMM, *Eminentia medialis*; GD, *Gyrus dentatus*; HIPP, hippocampal field; HYP, *Hypothalamus*; MA, matrix; MES, *Mesencephalon*; NC, *Nucleus caudatus*; Ntri, *Nucleus trigeminalis*; OB, olfactory bulb; PC, *Plexus choroideus*; Po, *Pons*; Sep, *Septum*; SepMA, matrix of the septum; StMed, *Striae medullares*; Str. rad., *Stratum radiatum lacunosum moleculare* of the hippocampus; STR, *Striatum*; SUB, *Subiculum*; TH, *Thalamus.*
(PDF)

Figure S2 Clustergrams/heat maps for all 33 nuclear encoded subunits of complex I during pre- and postnatal murine development. The abbreviations of the brain structures correspond to those detailed on Figure S1.
(PDF)

File S1 Heat maps depicting the intensity distribution of Z-transformed intensities with spatial resolution for all single probes during all investigated stages of pre- and postnatal development.
(PDF)
