# Supplementary figures and images for: Region-Specific Expression of Mitochondrial Complex I Genes during Murine Brain Development
Source: PLoS One. 2011 Apr 27;6(4):e18897. doi: 10.1371/journal.pone.0018897 (PMC3083399; doi:10.1371/journal.pone.0018897)

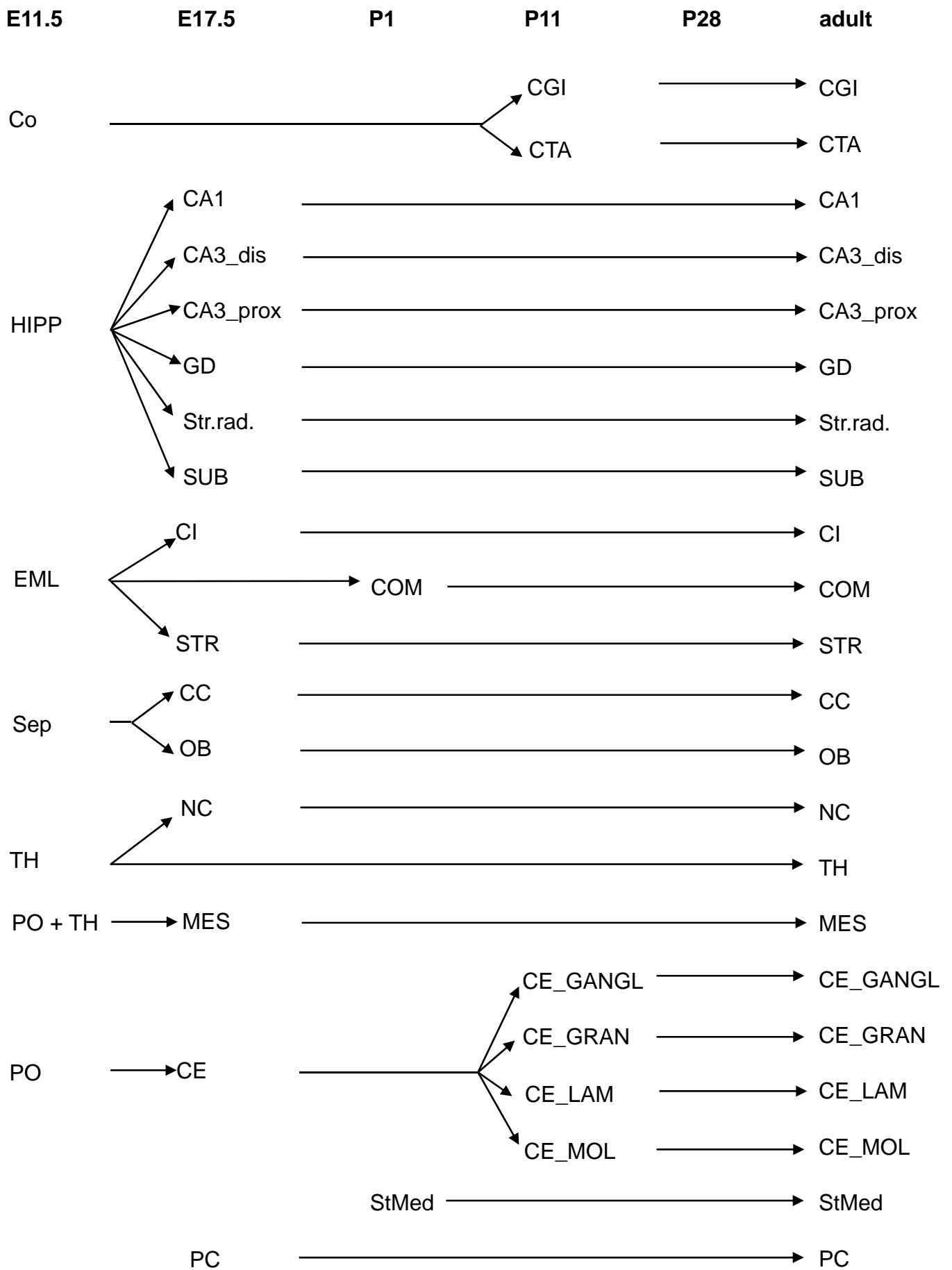

Supplement: Figure S1 — Investigated brain structures at the various time point of murine brain development. Abbreviations (in alphabetic order): CA1, CA1 region; CA3_dis, distal part of the CA3 region; CA3_prox, proximal part of the CA3 region; CC, Corpus callosum; CI, Capsula interna; CGI, cortex-granular insular; CE, cerebellar field; CE_GANGL, Cerebellum stratum ganglionare; CE_GRAN, Cerebellum stratum granulosum; CE_LAM, Cerebellum laminae medullares; CE_MOL, Cerebellum stratum moleculare; Co, cortex; COM, Commissura anterior; CTA, cortex-temporal anterior; EML, Eminentia lateralis; EMM, Eminentia medialis; GD, Gyrus dentatus; HIPP, hippocampal field; HYP, Hypothalamus; MA, matrix; MES, Mesencephalon; NC, Nucleus caudatus; Ntri, Nucleus trigeminalis; OB, olfactory bulb; PC, Plexus choroideus; Po, Pons; Sep, Septum; SepMA, matrix of the septum; StMed, Striae medullares; Str. rad., Stratum radiatum lacunosum moleculare of the hippocampus; STR, Striatum; SUB, Subiculum; TH, Thalamus. (PDF) [file pone.0018897.s001.pdf]

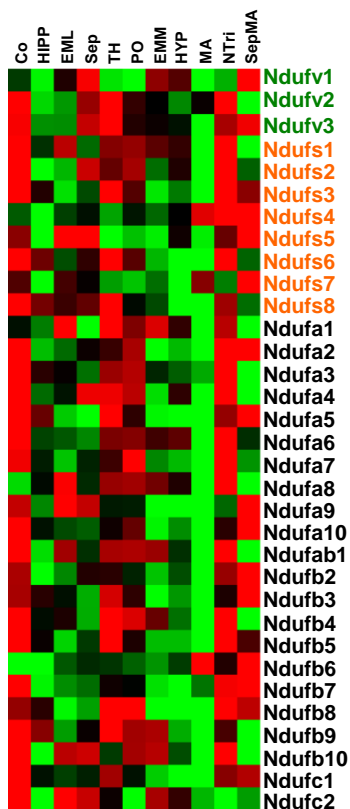

E11.5

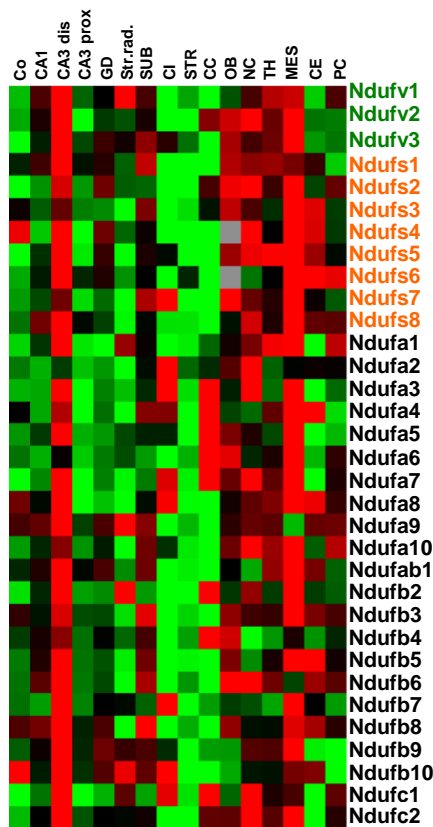

E17.5

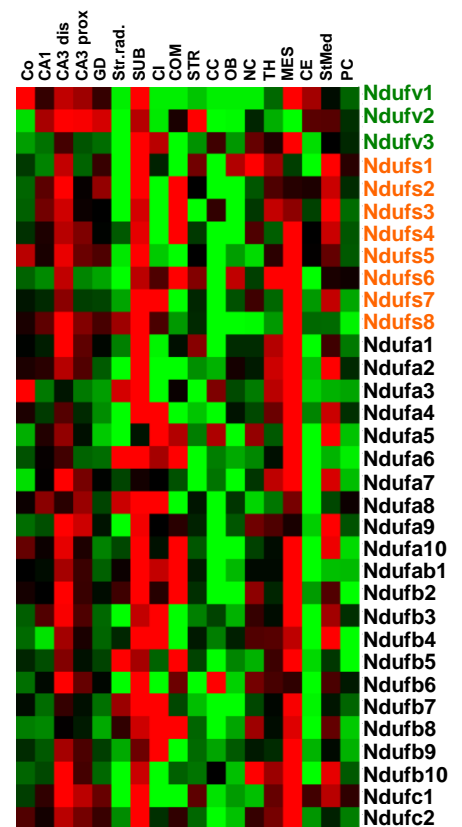

P1

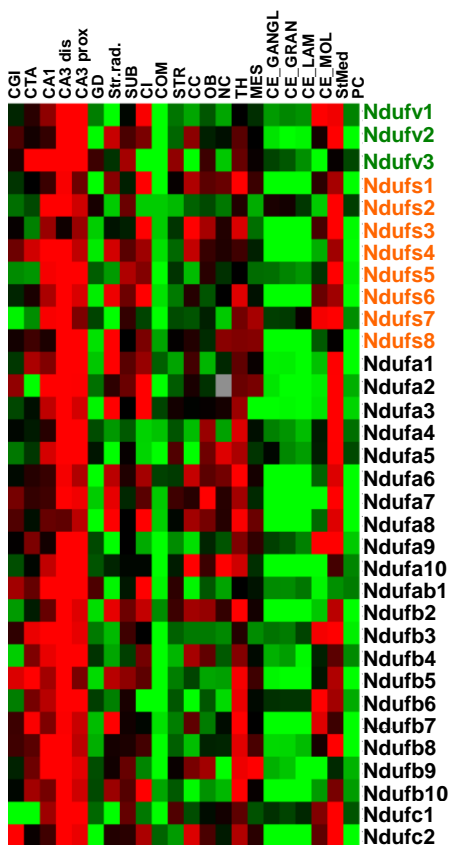

P11

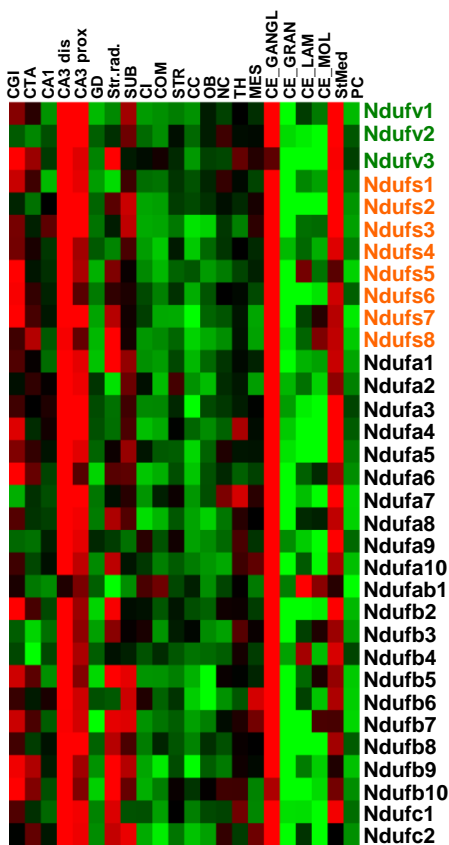

P28

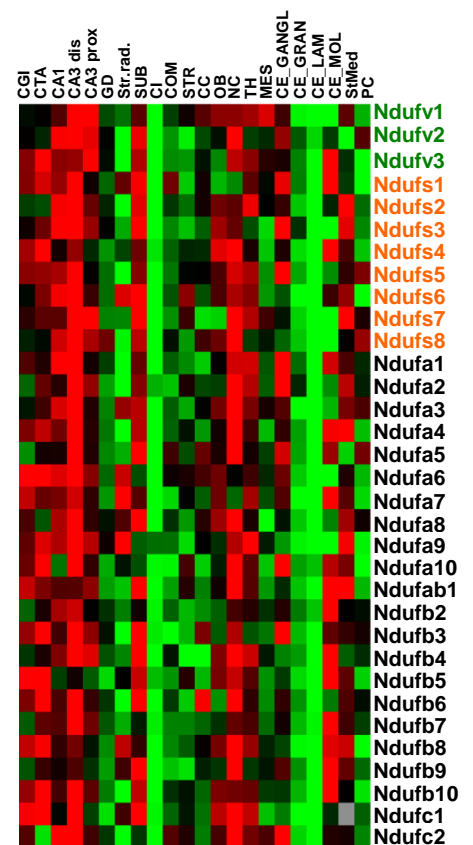

adult

Supplement: Figure S2 — Clustergrams/heat maps for all 33 nuclear encoded subunits of complex I during pre- and postnatal murine development. The abbreviations of the brain structures correspond to those detailed on Figure S1. (PDF) [file pone.0018897.s002.pdf]

# Embryonic day E11.5

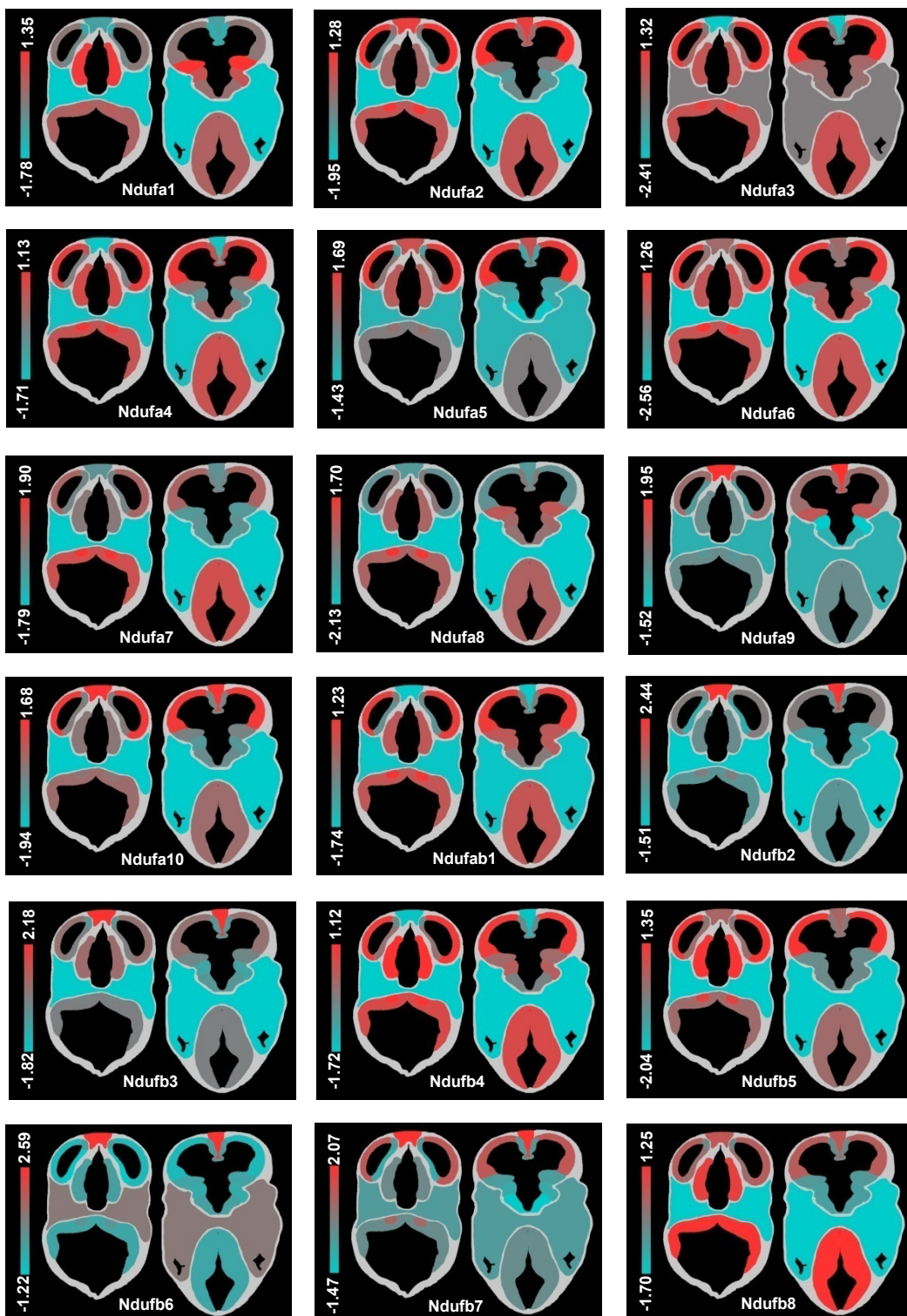

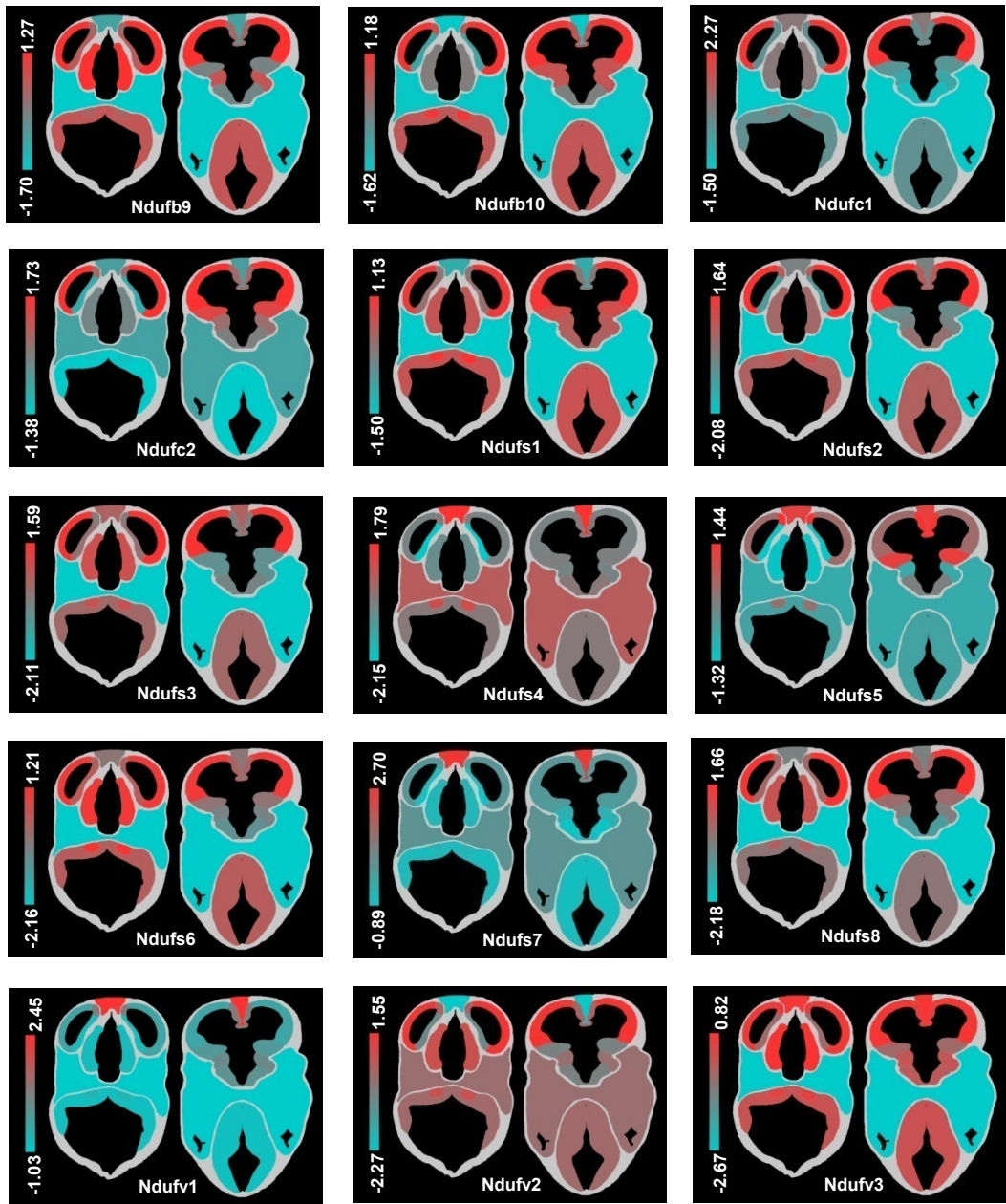

## Embryonic day E17.5

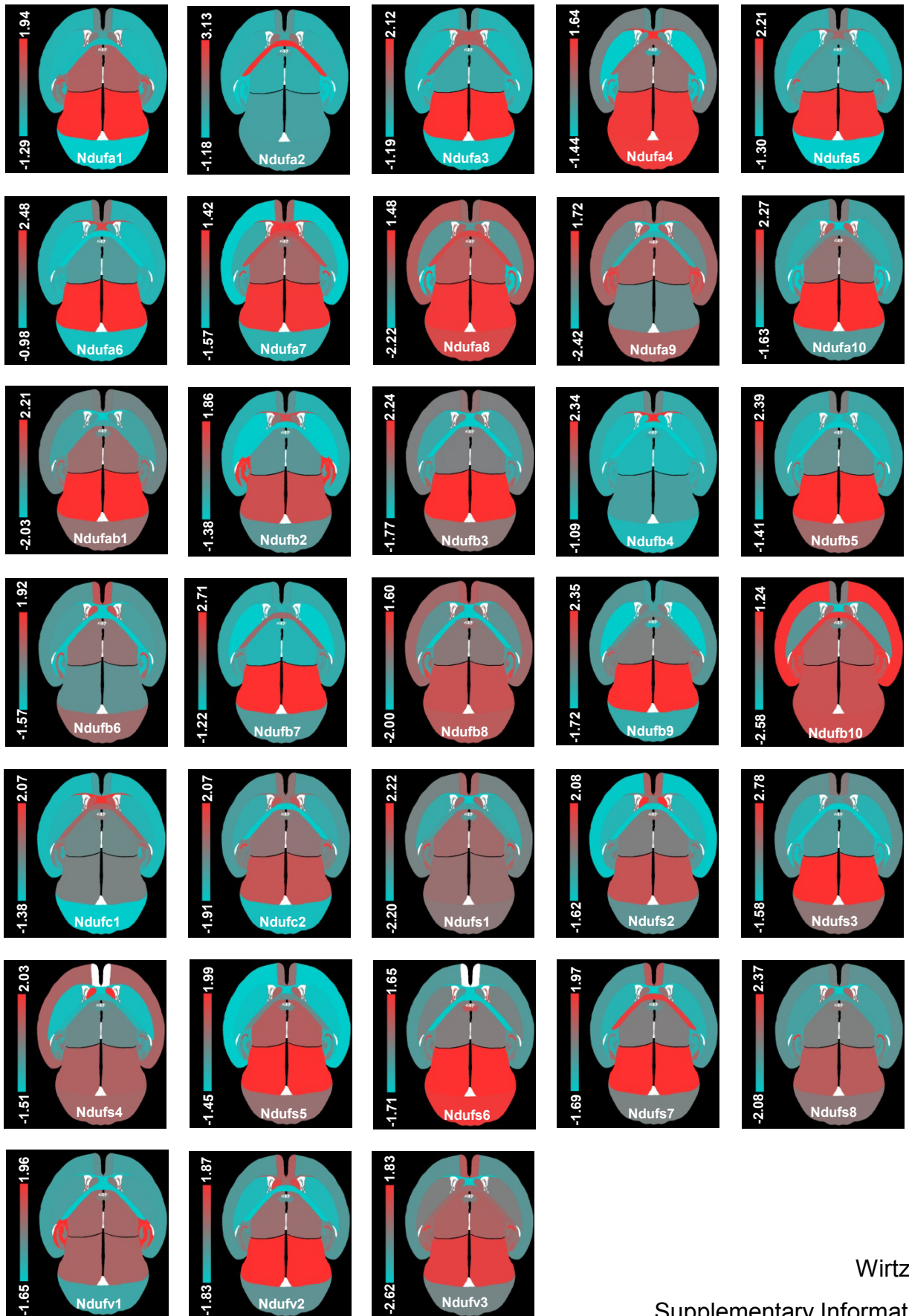

# Postnatal day P1

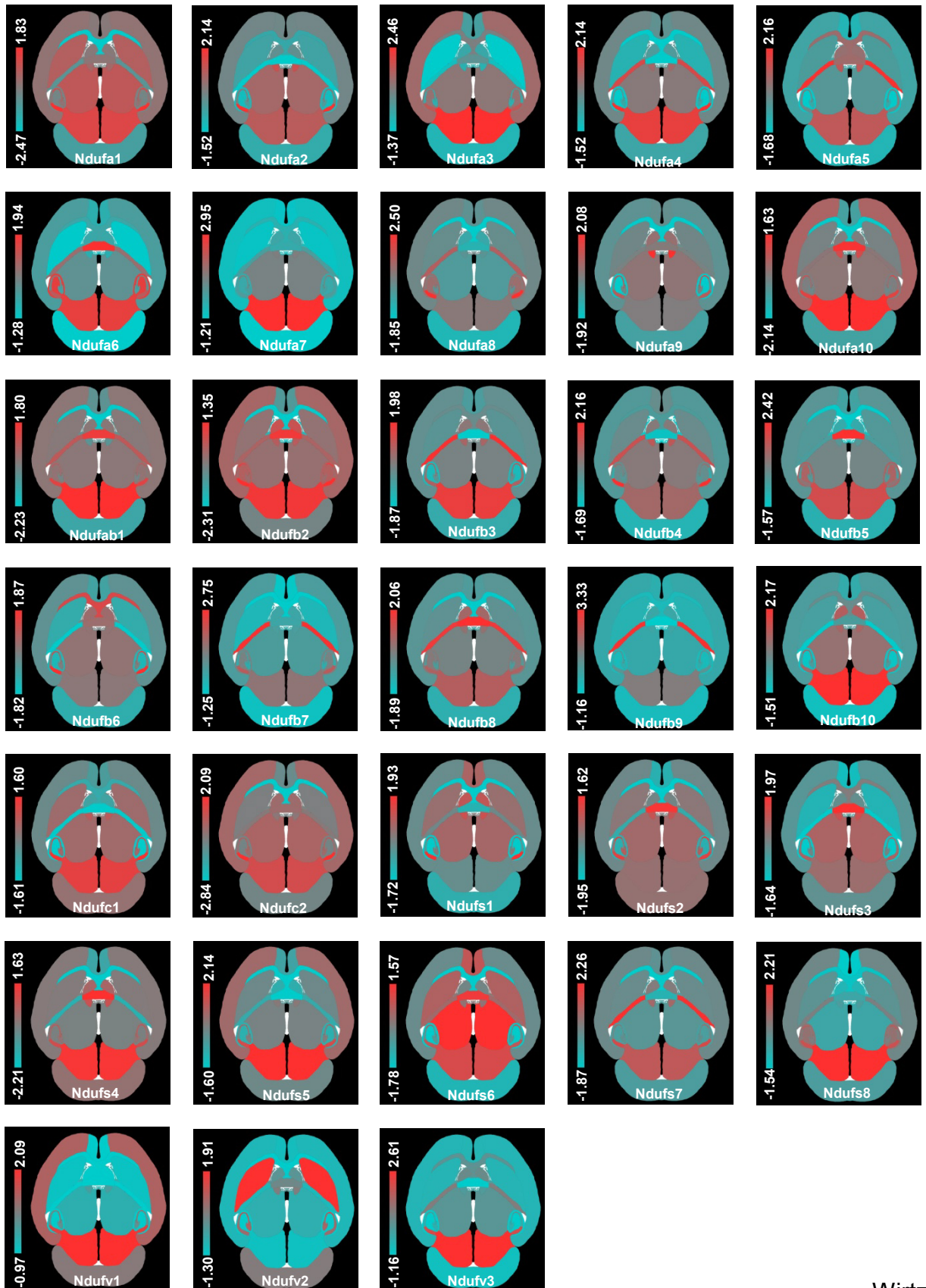

# Postnatal day P11

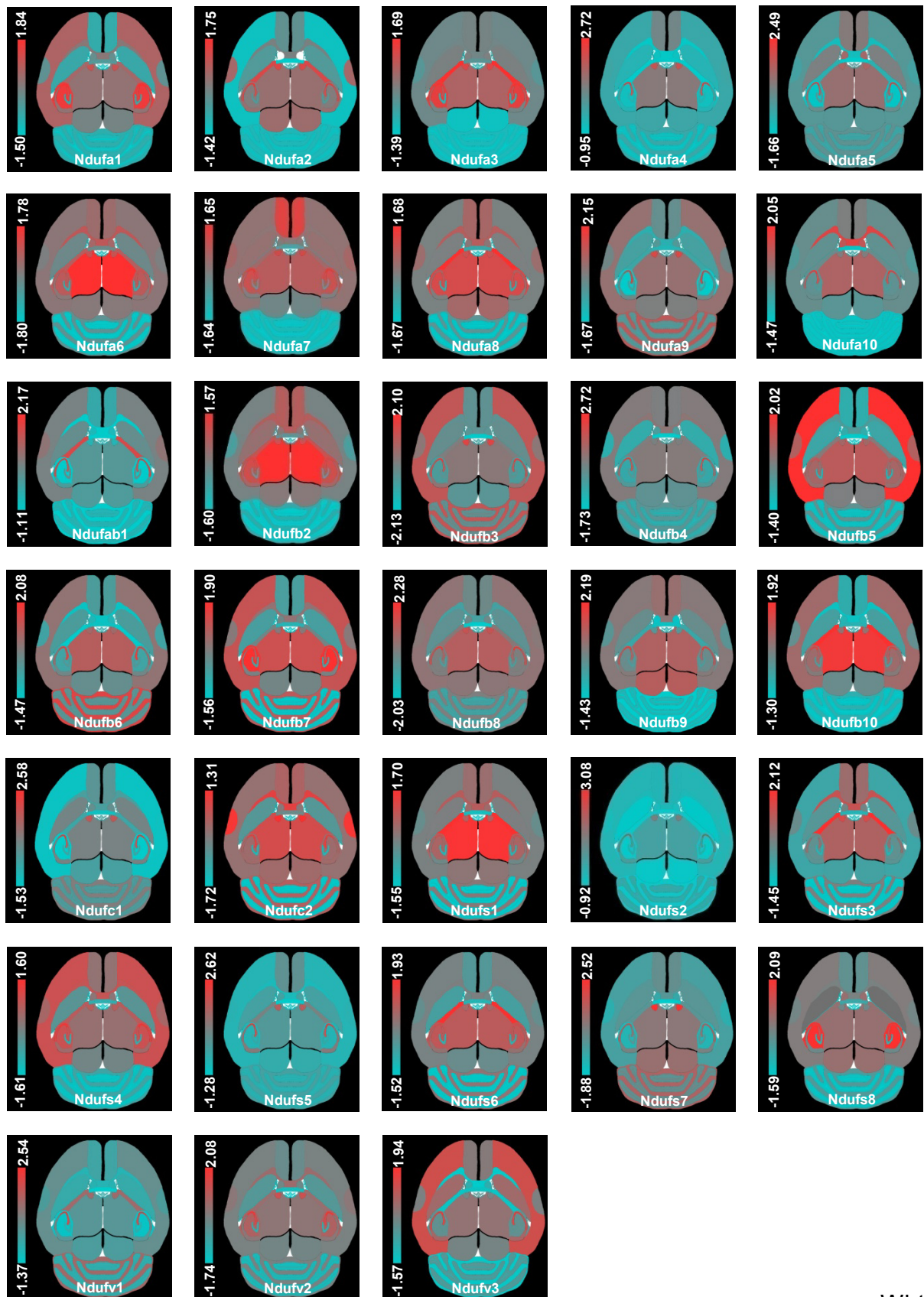

# Postnatal day P28

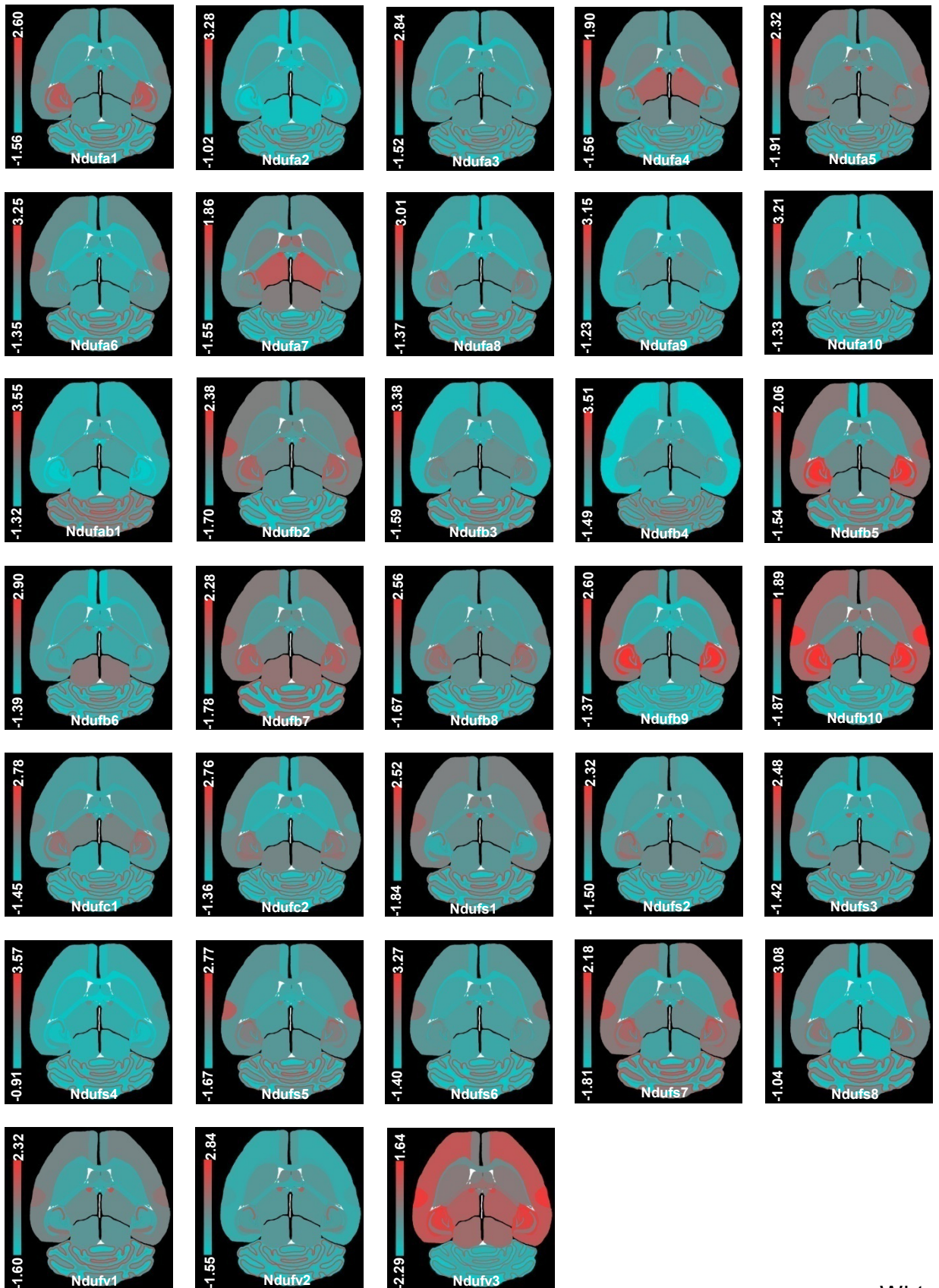

# Adult (12 weeks)

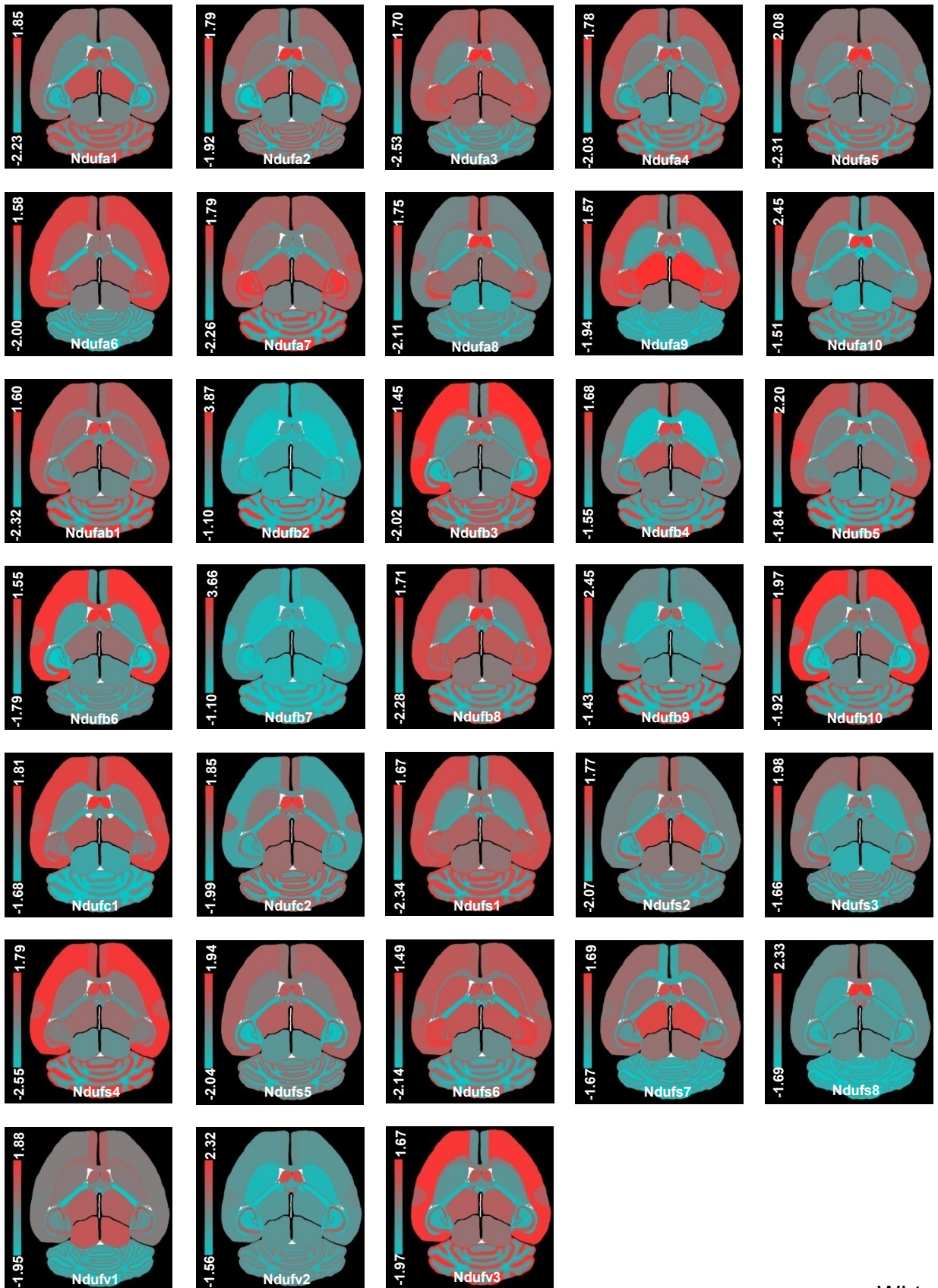

Supplement: File S1 — Heat maps depicting the intensity distribution of Z-transformed intensities with spatial resolution for all single probes during all investigated stages of pre- and postnatal development. (PDF) [file pone.0018897.s003.pdf]
